# Macrophages in spatial context: cellular niches in lung disease

**DOI:** 10.3389/fimmu.2026.1977224

**Published:** 2026-10-06

**Authors:** Akira Yamamoto, Nobuharu Fujii, Yoshinobu Maeda

**Affiliations:** 1 Department of Hematology and Oncology, Okayama University Hospital, Okayama, Japan; 2 Dan L Duncan Comprehensive Cancer Center, Baylor College of Medicine, Houston, TX, United States; 3 Division of Transfusion and Cell Therapy, Okayama University Hospital, Okayama, Japan

**Keywords:** bronchiolitis obliterans, lung macrophages, macrophage niche, monocyte-derived macrophages, pathological cellular niche, pulmonary fibrosis, spatial omics, tissue repair

## Abstract

Advances in spatial omics are shifting the study of lung macrophages from the characterization of isolated transcriptional states toward an understanding of their function in the context of cellular origin, anatomical location, neighboring cells, and tissue architecture. Here, we distinguish the macrophage niche in the strict sense—the local microenvironment that sustains tissue-resident macrophage survival and identity—from the broader cellular niche, in which macrophages are spatially organized with multiple interacting cell populations, and integrate recent insights into lung repair and chronic remodeling. Following injury, remodeling of the resident macrophage compartment, recruitment of monocyte-derived macrophages, and the emergence of regenerative epithelial intermediates and stromal responses generate transient multicellular environments that normally resolve as tissue homeostasis is restored. Spatial analyses, however, indicate that in at least some lung diseases, these repair-associated states fail to resolve and instead become organized into pathological cellular niches characterized by recurrent proximity among aberrant epithelial cells, inflammatory and profibrotic macrophages, activated stromal cells, and extracellular matrix. We further propose a spatial framework in which shared immune–epithelial–stromal interactions can give rise to distinct fibrotic architectures depending on whether they are anchored within alveolar–interstitial regions or around small airways. Finally, we discuss how pathological cellular niches identified by spatial approaches can be functionally validated and translated into therapeutically actionable intercellular circuits.

## 1 Introduction

### 1.1 Spatial organization of the lung and the limitations of dissociated profiling

The lung is a highly compartmentalized organ composed of alveolar, airway, vascular, and interstitial regions, and cellular function is determined not only by molecular state but also by anatomical location and the composition of the surrounding cellular microenvironment. Traditionally, the spatial organization of pulmonary lesions has been characterized by combining organ-scale imaging, including chest radiography and computed tomography (CT), with tissue- and cellular-scale analyses by histopathology and immunohistochemistry. Radiomics, digital pathology, and highly multiplexed imaging have further advanced the quantitative assessment of morphological features and spatial relationships among cells (1, 2). However, these approaches have remained limited in their ability to comprehensively map tissue morphology and molecular states of individual cells within a common spatial coordinate system (3).

Single-cell RNA sequencing (scRNA-seq) has enabled comprehensive cataloguing of the diverse cell types and cellular states that constitute the lung (4). Conventional scRNA-seq, however, requires tissue dissociation, thereby erasing information about the anatomical location of individual cells and their relationships with neighboring cells (3). This limitation is particularly important for macrophages, whose functional states are highly responsive to local microenvironmental cues, and has hindered efforts to define their functional roles in disease.

To overcome this limitation, spatial profiling technologies that capture molecular information while preserving tissue architecture have rapidly advanced (**Table 1**) (5, 6). By linking cellular states to anatomical location, tissue architecture, and neighboring cell populations, these approaches restore a dimension of biological information that is lost during conventional single-cell profiling. In a highly compartmentalized organ such as the lung, this spatial perspective provides a framework for viewing disease not simply as a quantitative shift in cellular composition, but as the emergence, persistence, or reorganization of local multicellular structures within defined anatomical contexts. Consistent with this concept, recent spatial atlases of the human lung have demonstrated regional variation in cellular composition and transcriptional programs across anatomical compartments while also identifying recurrent cellular neighborhoods and localized immune niches (7, 8).

**Table 1:** Major spatial profiling modalities for investigating macrophage-centered cellular niches in the lung.

Modality	Representative platforms	Primary readout	Main strength	Major limitation	Ref.
ROI-based profiling	GeoMx DSP	High-plex RNA and/or protein abundance within selected ROIs	Pathology-guided profiling of defined tissue regions	Regional averaging and ROI-selection bias	(14)
Capture-based spatial transcriptomics	ST/Visium, Visium HD, Slide-seq	Spatially barcoded whole-transcriptome RNA counts	Unbiased discovery of spatial gene-expression domains	Resolution–sensitivity trade-off and potential cellular mixing	(15–17)
Image-based spatial transcriptomics	MERFISH, seqFISH+, Xenium, CosMx	Spatial coordinates of predefined RNA molecules; RNA and protein on selected platforms	Single-cell and subcellular localization with direct neighborhood analysis	Panel dependence and segmentation errors	(18–21)
Spatial multi-omics sequencing	DBiT-seq	Co-mapped mRNA and antibody-derived protein signals	Simultaneous measurement of multiple molecular modalities	Pixel-level cellular mixing and technical complexity	(22)
Multiplex protein imaging	IMC, MIBI/MIBI-TOF, CODEX	Antibody-defined proteins and protein modifications	*In situ* cell phenotyping and spatial proximity analysis	Dependence on antibody panels and cell segmentation	(2, 23, 24)
Mass spectrometry imaging	MALDI-MSI and related platforms	Spatial distributions of molecular ions, including metabolites, lipids and glycans	Broad chemical mapping without predefined molecular panels	Molecular annotation and cell-type assignment remain challenging	(25, 26)

Representative spatial profiling modalities, their primary readouts, strengths, limitations, and key references are summarized.

CODEX, co-detection by indexing; DBiT-seq, deterministic barcoding in tissue for spatial omics sequencing; DSP, digital spatial profiling; IMC, imaging mass cytometry; MALDI-MSI, matrix-assisted laser desorption/ionization mass spectrometry imaging; MERFISH, multiplexed error-robust fluorescence *in situ* hybridization; MIBI-TOF, multiplexed ion beam imaging by time of flight; ROI, region of interest; ST, spatial transcriptomics.

### 1.2 Macrophage niches and pathological cellular niches

This spatial perspective is particularly important for understanding the functional heterogeneity of lung macrophages. Alveolar macrophages (AMs) and interstitial macrophages (IMs) constitute the major tissue-resident macrophage populations in the lung, whereas during injury, circulating monocytes can infiltrate the tissue, differentiate into macrophages, and in some settings persist long term (9, 10). These populations do not possess fixed functional identities but instead adapt their transcriptional and functional states in response to local signals from epithelial cells, fibroblasts, endothelial cells, lymphocytes, and the extracellular matrix (ECM). The concept of the “macrophage niche,” proposed by Guilliams and colleagues, posits that the maintenance and tissue-specific identity of resident macrophages are determined by the surrounding microenvironment, which provides physical scaffolds, survival factors, and intercellular signals (11). In this Review, we use *macrophage niche* in this strict sense to denote the local environment that supports the survival, maintenance, and tissue-specific identity of resident macrophages. By contrast, we use the broader term *cellular niche* to describe local structures in which macrophages and multiple other cell populations are spatially organized within a defined anatomical region, with potential or demonstrated intercellular interactions depending on the available evidence, and *pathological cellular niche* when such structures are associated with disease. Although these concepts may overlap, they are not synonymous. Within this framework, lung pathology may be shaped not only by changes in the abundance of particular macrophage populations but also by disruption and reorganization of pre-existing macrophage niches after injury and by the local cellular communities formed by newly recruited or persisting macrophages with surrounding cells (9–11). In particular, loss of resident macrophages followed by repopulation with monocyte-derived cells may represent a critical transition that influences whether the injured microenvironment returns to homeostasis or evolves into a persistent pathological multicellular state (**Figure 1**).

**Figure 1:**
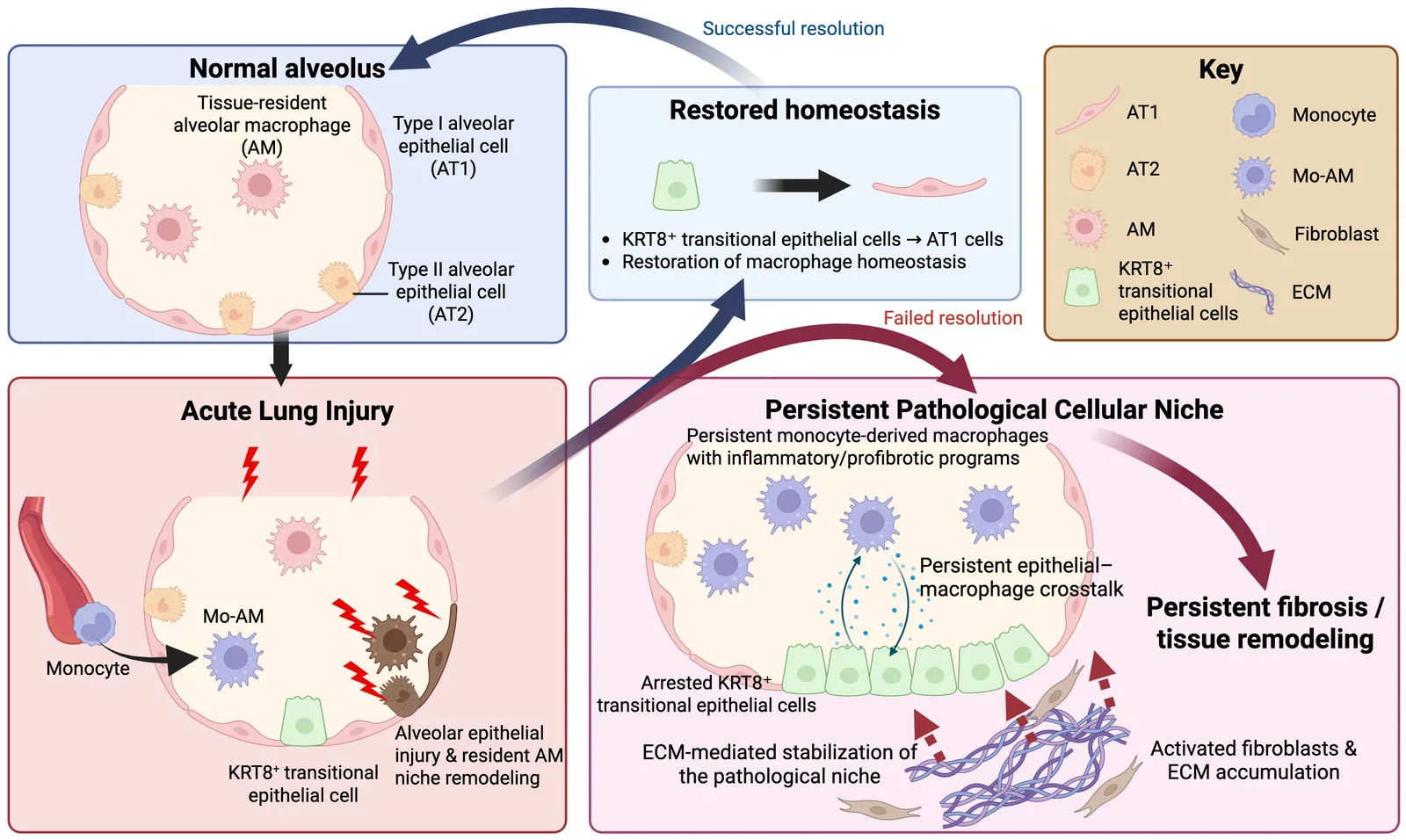
Conceptual model of successful alveolar repair and failed resolution leading to a persistent pathological cellular niche. In the normal alveolus, type I alveolar epithelial (AT1) and type II alveolar epithelial (AT2) cells form the alveolar epithelium, while tissue-resident alveolar macrophages (AMs) occupy the alveolar space. Acute lung injury damages the alveolar epithelium and remodels the resident AM niche, accompanied by recruitment of circulating monocytes and the emergence of KRT8^+^ transitional epithelial cells. During successful resolution, KRT8^+^ transitional epithelial cells differentiate into AT1 cells and macrophage homeostasis is restored, leading to recovery of tissue homeostasis. In contrast, failed resolution is characterized by persistent KRT8^+^ transitional epithelial cells with arrested differentiation and monocyte-derived macrophages with inflammatory and profibrotic programs. Persistent epithelial–macrophage crosstalk, together with fibroblast activation and extracellular matrix (ECM) accumulation, contributes to the establishment and maintenance of a persistent pathological cellular niche, ultimately promoting persistent fibrosis and tissue remodeling. ECM accumulation and remodeling may further stabilize pathological cellular relationships within the niche, thereby reinforcing its persistence. Dashed arrows indicate proposed ECM-mediated feedback relationships. The alveolar compartment is shown as a representative model of tissue repair and failed resolution; the cellular composition and resulting fibrotic architecture of pathological niches may vary according to anatomical context, including alveolar–interstitial and small-airway regions. Created in BioRender. Yamamoto, A (2026). https://BioRender.com/mo2ohpt. AM, alveolar macrophage; AT1, type I alveolar epithelial cell; AT2, type II alveolar epithelial cell; ECM, extracellular matrix; KRT8, keratin 8; Mo-AM, monocyte-derived alveolar macrophage.

Recent spatial studies support this concept. Following acute lung injury, regenerative epithelial intermediates and inflammatory macrophages can form localized cellular communities, whereas in chronically fibrotic lungs, aberrant epithelial cells, macrophages, and activated stromal cells repeatedly accumulate within defined tissue regions (12, 13). These observations suggest that failure to resolve normally transient repair-associated cellular states and interactions may contribute to the establishment of pathological niches that sustain chronic tissue remodeling. Moreover, similar macrophage–epithelial–stromal interactions may produce distinct pathological outcomes depending on their anatomical context: when organized within alveolar–interstitial regions, they may contribute to destruction of alveolar architecture, whereas when anchored around small airways, they may promote airway narrowing and obliteration. Understanding pulmonary fibrosis therefore requires consideration not only of pathological cellular states themselves, but also of the anatomical compartments in which they persist and the cellular relationships that sustain them.

### 1.3 Conceptual framework and scope of this review

In this Review, we use the concept of failed resolution of the repair niche as a framework for integrating spatial evidence across defective repair after acute lung injury and pathological cellular niches in chronic fibrotic lung disease. We first briefly outline the spatial profiling technologies and integrative approaches used to resolve these cellular niches in intact lung tissue. We then focus on disruption of resident macrophage niches and their replacement by monocyte-derived populations after injury, persistence of regenerative epithelial intermediate states, and stabilization of fibrotic niches through macrophage–epithelial–fibroblast interactions (**Table 2**). We further compare the predominantly alveolar–interstitial remodeling of idiopathic pulmonary fibrosis (IPF) with the airway-centered remodeling observed in bronchiolitis obliterans (BO) after hematopoietic stem cell transplantation and chronic lung allograft dysfunction (CLAD) after lung transplantation, asking how shared cellular interactions can generate distinct fibrotic architectures when organized on different anatomical scaffolds. Finally, by comparing these conditions with other diseases characterized by macrophage-centered spatial organization, we consider the generalizability and limitations of this niche-based model and its potential to identify therapeutically actionable intercellular circuits.

**Table 2:** Representative macrophage-associated cellular niches in lung disease.

Model/anatomical location	Macrophage population/state	Nearby cells/proposed interaction	Methods	Functional/mechanistic assessment	Ref.
Viral lung injury					
Mouse/influenza A (H1N1, PR8); dysplastic alveolar repair regions	Monocyte-derived macrophages	Persistent CD8+ T cells, dysplastic epithelial progenitors; persistent CD8+ T cells sustain IL-1β-producing macrophages through IFN-γ/TNF signaling, impairing epithelial differentiation and alveolar regeneration	Visium spatial transcriptomics; immunofluorescence imaging	IFN-γ/TNF or IL-1β blockade; alveolar regeneration and lung function assessed	(12)
Mouse/influenza A (H1N1, PR8); alveolar compartment during epithelial repair	Plet1+ regenerative BMDMs and TR-AMs	Alveolar epithelial cells and progenitors; macrophage-derived Plet1 promotes epithelial survival, progenitor proliferation, alveolarization, and barrier repair	scRNA-seq; histology; immunofluorescence imaging	Plet1 neutralization and BMDM-specific Plet1 deletion; epithelial proliferation, organoid growth, and barrier repair assessed	(42)
Human/COVID-19 DAD; alveolar regions across DAD stages	SPP1-expressing macrophages enriched in EDAD; alveolar and CHIT1+ macrophages increasing with DAD progression	ECs, epithelial cells, fibroblasts; macrophage-derived SPP1/OPN may promote a prothrombotic and antifibrinolytic EC state through SERPINE1/PAI-1 induction in EDAD	GeoMx DSP/WTA; Visium; smFISH; integrated sc/snRNA-seq	Recombinant OPN stimulation of ECs; PAI-1 induction assessed	(76)
Pulmonary fibrosis					
Mouse/bleomycin; alveolar compartment during fibrosis and resolution	Mo-AMs	Not specifically resolved; Mo-AMs, but not TR-AMs, promote fibrosis and persist after resolution while undergoing progressive transcriptional differentiation	Genetic lineage tracing; flow cytometry; bulk RNA-seq; histology	Selective genetic depletion of Mo-AMs; fibrosis assessed	(44)
Mouse/asbestos; fibrotic regions near asbestos fibers at bronchoalveolar duct junctions	Mo-AMs	Fibroblasts, injured epithelial cells; M-CSF/M-CSFR signaling sustains Mo-AMs within fibrotic niches, where PDGFA-expressing Mo-AMs localize near fibroblasts	scRNA-seq; smFISH; genetic lineage tracing; immunofluorescence imaging	Genetic depletion of Mo-AMs and pharmacological M-CSFR blockade; fibrosis assessed	(39)
Human/IPF; remodeled airways and adjacent airway lumen	SPP1+ airway macrophages	Aberrant basaloid cells, myofibroblasts, airway epithelial cells; an SPP1+ macrophage-rich airway niche occupies the airway lumen adjacent to a fibrotic niche containing aberrant basaloid cells and myofibroblasts	Visium FFPE/CytAssist; Xenium *in situ*; COMET high-plex sequential immunofluorescence; integrated scRNA-seq	Niche organization validated by Xenium and high-plex protein imaging; spatially informed ligand–receptor analysis performed	(40)
Human/PF across multiple ILD diagnoses; distal airspaces across remodeling severity	FABP4+ alveolar macrophages and SPP1+ macrophages	Alveolar epithelial cells, transitional epithelial cells, fibroblasts; macrophage composition and localization vary with distal-airspace remodeling, with SPP1+ macrophages becoming more prominent with increasing pathology	Xenium *in situ*; Visium HD; H&E image registration; cell-neighborhood and trajectory analyses	Cross-sectional airspaces ordered by molecular pseudotime; matched Visium HD used to confirm macrophage accumulation patterns	(13)
BOS/CLAD					
Human/lung transplantation (stable, BOS, RAS); alveolar compartment	Donor-derived metabolically adapted AMs in stable grafts; recipient-derived inflammatory AMs in CLAD	Not specifically resolved; stable long-term grafts retain donor-derived AMs, whereas CLAD is associated with their replacement by recipient-derived inflammatory macrophages	Spatial transcriptomics; X/Y FISH	AM origin determined by X/Y FISH; stable grafts, BOS, and RAS compared for macrophage origin and polarization	(53)
Human/post-HSCT BO; within and around obliterated bronchioles	Foamy CD68+CD206+ macrophages and CD68+CD206+α-SMA+ cells	Bronchiolar epithelial cells, myofibroblasts, fibrotic stroma; foamy macrophages show stage- and region-dependent shifts from inflammatory to fibrotic programs, with marker coexpression compatible with MMT	GeoMx DSP; multiplex immunofluorescence	Stage- and location-dependent transcriptional profiling; CD68/CD206/α-SMA coexpression examined	(64)
Others					
Human/chronic pulmonary sarcoidosis; granuloma core and surrounding peripheral regions	SPP1+CHIT1+CHI3L1+ granuloma-core macrophages	CD3+ T cells, CD20+ B cells, COL3A1+/CTHRC1+ fibroblasts; a hybrid proinflammatory and profibrotic macrophage core is spatially organized with adjacent lymphocyte niches and a peripheral fibroblast niche	Visium spatial transcriptomics; RNA *in situ* hybridization; immunohistofluorescence	RNA and protein *in situ* validation; candidate inter-niche signaling examined by ligand–receptor analysis	(67)
Human + mouse/NSCLC; tumor-cell-proximal regions in early lesions	Tissue-resident macrophages	Tumor cells, Treg, CD8+ T cells; tissue-resident macrophages promote tumor-cell EMT and invasiveness and support Treg-mediated immune suppression during early tumor development	scRNA-seq; genetic lineage tracing; confocal microscopy	Tissue-resident macrophages depletion; tumor growth and invasiveness and Treg/CD8+ T-cell responses assessed	(69)
Human + mouse/NSCLC; SLC2A1+ TAM-rich tumor regions	SLC2A1+ TAMs	CD8+ T cells; SLC2A1+ TAMs promote spatial exclusion and functional impairment of CD8+ T cells and are associated with resistance to anti-PD-(L)1 therapy	Spatial transcriptomics; multiplex immunofluorescence; dual immunohistochemistry	TAM-specific Slc2a1 deletion; CD8+ T-cell distribution and function, tumor growth, and anti-PD-L1 response assessed	(71)

Representative studies are summarized according to disease or model, species, anatomical location, macrophage population or state, nearby cell types, proposed interactions, spatial or localization methods, and functional or mechanistic assessments, including the experimental approaches used to examine candidate mechanisms.

AM, alveolar macrophage; BMDM, bone marrow-derived macrophage; BO, bronchiolitis obliterans; BOS, bronchiolitis obliterans syndrome; CHIT1, chitinase 1; CHI3L1, chitinase 3-like 1; CLAD, chronic lung allograft dysfunction; COL3A1, collagen type III alpha 1 chain; CTHRC1, collagen triple helix repeat containing 1; DAD, diffuse alveolar damage; DSP, digital spatial profiling; EC, endothelial cell; EDAD, early diffuse alveolar damage; EMT, epithelial–mesenchymal transition; FABP4, fatty acid binding protein 4; FFPE, formalin-fixed paraffin-embedded; FISH, fluorescence *in situ* hybridization; H&E, hematoxylin and eosin; HSCT, hematopoietic stem cell transplantation; IFN-γ, interferon-γ; IL-1β, interleukin-1β; ILD, interstitial lung disease; IPF, idiopathic pulmonary fibrosis; M-CSF, macrophage colony-stimulating factor; M-CSFR, macrophage colony-stimulating factor receptor; MMT, macrophage-to-myofibroblast transition; Mo-AM, monocyte-derived alveolar macrophage; NSCLC, non-small-cell lung cancer; OPN, osteopontin; PAI-1, plasminogen activator inhibitor-1; PD-1, programmed cell death protein 1; PD-L1, programmed death-ligand 1; PDGFA, platelet-derived growth factor subunit A; PF, pulmonary fibrosis; Plet1, placenta-expressed transcript 1; RAS, restrictive allograft syndrome; scRNA-seq, single-cell RNA sequencing; SERPINE1, serpin family E member 1; SLC2A1, solute carrier family 2 member 1; smFISH, single-molecule fluorescence *in situ* hybridization; snRNA-seq, single-nucleus RNA sequencing; SPP1, secreted phosphoprotein 1; TAM, tumor-associated macrophage; TNF, tumor necrosis factor; TR-AM, tissue-resident alveolar macrophage; Treg, regulatory T cell; WTA, whole transcriptome atlas; α-SMA, alpha-smooth muscle actin.

## 2 Spatial technologies and integrative approaches for resolving macrophage niches

While conventional histological and imaging analyses preserve tissue architecture to assess cellular localization, they remain limited in their capacity for comprehensive molecular profiling. Conversely, dissociation-based approaches such as scRNA-seq provide high-dimensional molecular insights at the expense of native spatial context (3, 6). To bridge these complementary trade-offs, spatial profiling technologies have emerged to simultaneously capture molecular profiles and tissue architecture within the same spatial coordinate system (**Table 1**).

These approaches include region-of-interest (ROI)-based profiling, such as GeoMx Digital Spatial Profiling (DSP), which enables molecular analysis of pathologically selected tissue regions (14); capture-based spatial transcriptomic platforms, including ST/Visium, Visium HD, and Slide-seq, which provide broad spatially resolved gene-expression measurements (15–17); and image-based spatial transcriptomic technologies, including multiplexed error-robust fluorescence *in situ* hybridization (MERFISH), sequential fluorescence *in situ* hybridization (seqFISH+), Xenium, and CosMx (18–21). These approaches involve distinct trade-offs among molecular coverage, spatial resolution, and cellular assignment. ROI-based methods are subject to signal averaging within selected regions, capture-based approaches can be affected by cellular mixing and uncertainty in assigning measurements to individual cells, and high-resolution image-based approaches generally depend on predefined molecular panels and accurate cell segmentation (3, 14, 16, 20, 21). Beyond transcriptomics, spatial multi-omic approaches such as deterministic barcoding in tissue sequencing (DBiT-seq) (22), multiplexed protein-imaging platforms including imaging mass cytometry (IMC), multiplexed ion beam imaging by time of flight (MIBI/MIBI-TOF), and co-detection by indexing (CODEX) (2, 23, 24), and mass spectrometry imaging, including matrix-assisted laser desorption/ionization mass spectrometry imaging (MALDI-MSI) (25, 26), provide complementary means of characterizing local tissue microenvironments across distinct molecular layers, including proteins, metabolites, and lipids.

Advances in spatial biology have been driven not only by measurement technologies but also by computational approaches that reconstruct cellular states and tissue organization from spatially resolved data. Methods such as Tangram and cell2location integrate the rich molecular information provided by scRNA-seq or snRNA-seq with spatial measurements by projecting transcriptionally defined cell types and states onto tissue space (27, 28). Beyond localization of individual cell states, analyses based on spatial neighborhood graphs and local cellular composition can identify recurrent cellular neighborhoods and microanatomical domains (29, 30). Computational inference of cell–cell communication has likewise evolved from ligand–receptor expression-based frameworks, exemplified by CellChat (31), toward approaches such as COMMOT and Spacia that directly incorporate spatial information into the inference of cell–cell communication (32, 33). These approaches can prioritize candidate cell–cell interactions by jointly considering molecular relationships and spatial context. However, inferred neighborhoods and communication networks represent spatial associations or computationally predicted interactions and do not, by themselves, establish functional communication or causality.

At single-cell resolution, the accuracy of spatial analysis also depends critically on how individual cells are delineated and transcripts are assigned to them. In subcellular-resolution spatial transcriptomic datasets, segmentation errors can result in the misassignment of transcripts between adjacent cells, thereby distorting inferred cellular states and propagating into downstream analyses, including differential expression, neighborhood-dependent expression analyses, and ligand–receptor-based inference of cell–cell interactions (34). Several computational approaches have therefore been developed to improve cell segmentation, including Baysor, which infers cellular boundaries using transcriptional composition and, when available, cell-morphology information (35); SCS, which integrates imaging and transcriptomic information using a transformer-based neural network (36); and BIDCell, a self-supervised deep-learning framework that integrates spatial gene expression, cellular morphology, and cell-type information, including single-cell transcriptomic reference data (37). Complementing these efforts to improve segmentation itself, Mitchel and colleagues showed that misassigned transcripts can persist after segmentation as molecular admixture and substantially distort downstream analyses; they further developed an approach based on matrix factorization of local molecular neighborhoods to identify and separate these mixed signals (34). Thus, increasingly accurate spatial analysis requires not only improved delineation of cellular boundaries but also recognition and correction of residual transcript misassignment after segmentation. Such computational refinement is essential for robust interpretation of cellular states and local intercellular relationships within complex tissues.

## 3 Macrophage interactions with neighboring cells within spatial lung niches

Lung macrophages do not function in isolation but shape local tissue environments through interactions with surrounding immune and structural cells. Advances in spatial transcriptomics and multiplexed imaging have enabled macrophage states to be analyzed in relation to anatomical location and neighboring-cell composition, revealing multicellular niches involving T cells, fibroblasts, neutrophils, epithelial cells, and other populations that can support either tissue repair or pathological remodeling.

Macrophage–T-cell interactions provide a clear example of how local immune circuits can influence tissue repair. Following viral pneumonia, Narasimhan and colleagues identified a spatially restricted immune–epithelial progenitor niche composed of tissue-persistent CD8^+^ T cells, macrophages, and epithelial progenitors with arrested differentiation (12). CD8+ T-cell-derived interferon-γ (IFN-γ) and tumor necrosis factor (TNF) sustained an inflammatory macrophage state, whereas macrophage-derived interleukin-1β (IL-1β) impaired normal epithelial progenitor differentiation. Blockade of IFN-γ/TNF or IL-1β improved alveolar regeneration and lung function, demonstrating that this T cell–macrophage–epithelial signaling circuit functionally contributes to persistent post-viral defective repair.

Macrophage–fibroblast interactions are particularly important in fibrotic remodeling. In murine pulmonary fibrosis, transitional and monocyte-derived alveolar macrophage populations localize within fibroblast-rich fibrotic regions and exhibit profibrotic activity (38). Joshi and colleagues further showed that monocyte-derived alveolar macrophages localize near fibroblasts within fibrotic lesions and express fibroblast growth-promoting factors, including PDGFA; depletion of these macrophages or inhibition of macrophage colony-stimulating factor (M-CSF)/M-CSFR signaling attenuated fibrosis (39). Consistent with these experimental findings, spatial studies of human IPF have revealed coordinated organization of macrophage, fibroblast/myofibroblast, and aberrant epithelial states within fibrotic tissue (13, 40).

Macrophages also participate in local inflammatory circuits involving neutrophils. In bleomycin-induced pulmonary fibrosis, Hiroki and colleagues showed that loss of transient receptor potential vanilloid 1 (TRPV1)+ nociceptor-mediated regulation increased vasoactive intestinal peptide (VIP) production by alveolar macrophages, promoting the accumulation of Siglec-F+ neutrophils and neutrophil extracellular trap formation through a TGF-β1-dependent pathway. VIP inhibition or hematopoietic cell-specific deletion of Vip reduced Siglec-F+ neutrophil accumulation and fibrosis (41). These findings provide functional evidence that macrophage-centered intercellular networks can extend to neutrophil-mediated tissue injury.

Macrophage–epithelial interactions can also support normal regeneration. Following influenza pneumonia, regenerative macrophage populations upregulated Plet1, and macrophage-derived Plet1 promoted alveolar epithelial cell proliferation and restoration of barrier integrity. Neutralization or conditional deletion of macrophage-derived Plet1 reduced epithelial progenitor proliferation and delayed alveolar barrier repair (42). Together with the pathogenic IL-1β-mediated circuit described above, these findings illustrate how macrophage interactions with neighboring cells can produce distinct regenerative or pathological outcomes according to local cellular context.

These intercellular relationships provide a mechanistic framework for the disease-specific repair and fibrotic niches discussed below.

## 4 Repair niches after acute lung injury

During normal repair after acute lung injury, macrophages contribute to distinct phases of tissue recovery by clearing damaged cells and cellular debris, promoting resolution of inflammation, and supporting epithelial regeneration, thereby facilitating restoration of tissue homeostasis (9). During this process, alveolar type 2 (AT2) cells differentiate into alveolar type 1 (AT1) cells through a transient regenerative intermediate state characterized by KRT8 expression (43). Under normal conditions, this transitional state resolves over time; however, in specific local microenvironments, differentiation can become arrested, resulting in defective repair (12).

A key component of this process is the injury-induced remodeling of the tissue-resident alveolar macrophage (AM) niche and the subsequent recruitment and persistence of monocyte-derived alveolar macrophages (Mo-AMs) (**Figure 1**). Whereas the maintenance and tissue-specific identity of resident macrophages are shaped by their surrounding niche, lung injury markedly alters the resident AM compartment and promotes the recruitment of circulating monocyte-derived cells into the tissue (9, 11). Misharin and colleagues showed that Mo-AMs recruited after lung injury can persist in the lung long term and transiently acquire a profibrotic transcriptional program during differentiation, including expression of *Pdgfa*, *Mmp12*, and *Mmp14* (44). Selective depletion of Mo-AMs attenuated pulmonary fibrosis, providing functional evidence that these cells promote fibrotic remodeling. Thus, the macrophage response to injury should not be viewed simply as transient inflammatory-cell infiltration, but rather as a process in which the pre-existing resident macrophage niche is reorganized and macrophages of distinct ontogenetic origins become incorporated into the newly established local microenvironment.

In parallel with these changes in the resident macrophage compartment, recent spatial studies indicate that local cellular niches formed by macrophages, other immune cells, and regenerating epithelial cells can influence the outcome of epithelial repair. As discussed above, post-viral lung injury can generate a spatially restricted niche containing persistent CD8+ T cells, inflammatory macrophages, and epithelial progenitors with arrested differentiation (12). Persistence of this multicellular circuit provides an example of how a normally transient repair environment can become stabilized into a pathological niche that prevents restoration of normal alveolar architecture.

A similar reorganization of epithelial–macrophage relationships after injury has been observed in severe human viral lung injury. Integrated histopathological, single-cell transcriptomic, and spatial transcriptomic analyses of COVID-19 lungs revealed stage-dependent changes in epithelial and macrophage states and their spatial distribution across the course of alveolar injury, with macrophage-associated signals including SPP1 linked to alveolar damage and tissue remodeling (45). Together, these findings are consistent with the possibility that defective repair after at least some forms of viral lung injury involves failure to resolve normally transient regenerative states and their local relationships with surrounding immune cells, although these spatial associations do not by themselves establish a causal mechanism. In particular, persistence of a multicellular signaling circuit involving CD8+ T cells, macrophages, and epithelial cells in regenerative intermediate states can transform a local cellular community that would normally support tissue repair into a pathological niche that sustains arrested epithelial differentiation (12). Understanding defective repair therefore requires consideration not only of individual cellular states, but also of the spatial relationships through which these states are reciprocally maintained.

Defective repair after acute lung injury, however, should not be viewed as progressing linearly into chronic pulmonary fibrosis. Pulmonary fibrosis arises from diverse etiologies and predisposing factors. Nevertheless, the concept that normally transient repair-associated states fail to resolve and that local cellular niches containing monocyte-derived macrophages persist provides a useful framework for understanding at least some forms of fibrotic disease (44, 46). In the next section, we examine the spatial niches that emerge in chronic pulmonary fibrosis through the lens of this failure to resolve the repair response.

## 5 Failed resolution of repair in fibrotic niches

The conceptual framework of “failed resolution of repair” introduced above provides a useful lens through which to interpret the persistent coexistence of aberrant epithelial states, macrophages, and stromal cells in chronic pulmonary fibrosis. The functional importance of monocyte-derived alveolar macrophages (Mo-AMs) in fibrotic remodeling is supported by bleomycin-induced pulmonary fibrosis models, in which Mo-AMs promote fibrosis and persist in the lung long after the initial injury (44). In human idiopathic pulmonary fibrosis (IPF), single-cell RNA sequencing (scRNA-seq) has identified a proliferative macrophage population expressing SPP1 and MERTK (47). In parallel, studies of the murine bleomycin model have identified transitional profibrotic macrophages with transcriptional features intermediate between monocytes and tissue-resident alveolar macrophages, highlighting the dynamic remodeling of macrophage states during fibrosis (38).

Spatial transcriptomic analyses are beginning to reveal how these pathological cell states are organized within fibrotic tissue. Mayr and colleagues integrated spatial transcriptomics of IPF and control lungs with an scRNA-seq atlas and identified multiple IPF-associated cellular niches (40). Among these, a fibrotic niche containing myofibroblasts and aberrant basaloid cells was found predominantly around remodeled airways, whereas an adjacent airway macrophage niche enriched in SPP1+ macrophages occupied nearby airway luminal regions. These spatial arrangements were further validated using Xenium and highly multiplexed protein imaging (40). Importantly, these findings argue against a model in which aberrant epithelial cells, fibroblasts, and macrophages are uniformly intermixed within a single homogeneous “fibrotic niche.” Rather, fibrotic tissue appears to be organized into distinct pathological niches with different cellular compositions that are spatially juxtaposed.

Vannan and colleagues further analyzed approximately 1.6 million cells from human lung tissue using Xenium and ranked distal lung airspaces along a molecular pseudotime continuum based on the proportion of transcripts corresponding to a healthy alveolar niche, enabling them to examine changes in cellular neighborhoods and cell states across a spectrum of fibrotic severity (13). Across relatively preserved to severely remodeled regions, they observed coordinated differences in molecular programs and cellular composition, beginning with abnormalities in the alveolar epithelium and extending to changes in macrophage polarization. Importantly, however, this analysis did not represent a longitudinal trajectory of the same lesion over time. Rather, it reconstructed a putative sequence of disease progression from distinct remodeling states coexisting within the same lung (13).

Taken together, these studies argue against viewing the fibrotic niche in IPF as a structure initiated and driven by macrophages alone. A more plausible model is that disruption of epithelial homeostasis and the emergence of aberrant regenerative states are accompanied by coordinated reorganization of macrophage and fibroblast states, potentially contributing to the establishment and persistence of a pathological local cellular environment (13, 40). In addition, ECM produced by activated fibroblasts is not merely an end product of fibrosis. By altering the physical and biochemical properties of the tissue microenvironment, the ECM may also provide a structural substrate that stabilizes these pathological cellular relationships (48–50). From this perspective, chronic pulmonary fibrosis can be viewed not simply as the emergence of disease-associated cell populations, but as the persistent spatial fixation of aberrant cellular states and their local relationships within tissue.

The anatomical sites at which this spatial fixation occurs, however, differ across diseases. In IPF, remodeling of the distal alveolar–interstitial lung is a central feature, yet spatial analyses also identify pathological fibrotic and macrophage niches around remodeled airways (13, 40). IPF should therefore not be reduced to a purely “alveolar-centered” fibrotic process, but rather viewed as heterogeneous remodeling distributed across multiple anatomical compartments. By contrast, in post-transplant obliterative bronchiolar disease, the small airway itself constitutes the principal anatomical scaffold for fibrotic remodeling. In the next section, we examine this distinction and consider how similar immune–epithelial–stromal interactions can generate different fibrotic architectures depending on the anatomical context in which they are organized.

## 6 Anatomical context and the architecture of fibrotic niches

Although the cellular programs associated with fibrosis show important commonalities across diseases, their ultimate histopathological consequences may be influenced by the anatomical compartment in which pathological cellular communities are established. As discussed above, airway-associated niches are also present in IPF; however, in post-transplant obliterative bronchiolar disease, fibrosis centered on the small-airway wall and its immediate surroundings is a defining pathological feature. This comparison raises the possibility that partially shared inflammatory, reparative, and fibrotic cellular programs can converge on distinct tissue architectures when organized on different anatomical scaffolds.

### 6.1 CLAD/BOS after lung transplantation

Chronic lung allograft dysfunction (CLAD) is an umbrella term describing a persistent decline in lung allograft function, and bronchiolitis obliterans syndrome (BOS) is one of its major clinical phenotypes (51). BOS is characterized predominantly by obstructive airflow limitation, with inflammation, fibrosis, and luminal obliteration of the small airways as its pathological substrate. Importantly, BOS is a clinical diagnosis based on pulmonary function and is not synonymous with histopathological bronchiolitis obliterans. Here, we focus on BOS as an airway-centered form of CLAD and examine how local cellular environments, including the macrophage compartment, are reorganized during disease.

Recent high-dimensional spatial studies are beginning to show that airway injury in CLAD is accompanied by coordinated changes in epithelial cells and immune-cell compartments. Imaging mass cytometry revealed a reduction in club cells and an increase in memory CD8+ T cells in CLAD lungs, together with spatial proximity between proliferative basal cells and memory T cells in restrictive allograft syndrome (RAS) (52). In parallel, lung transplantation provides a unique setting in which the long-term persistence and replacement of resident macrophage populations can be studied directly in humans. Schwarz and colleagues showed that donor-derived alveolar macrophages (AMs) with metabolic and tissue-homeostatic programs were relatively preserved in long-term stable allografts, whereas BOS and RAS were associated with loss of donor-derived AMs, replacement by recipient-derived macrophages, and a shift toward inflammatory polarization (53). These findings indicate that alterations in the airway epithelium and restructuring of the resident macrophage compartment coexist in CLAD. Whether macrophage replacement contributes causally to CLAD or instead reflects repeated allograft injury, however, remains unresolved.

These changes in the macrophage compartment occur within a broader tissue response involving the epithelium and vasculature. Spatial transcriptomic analysis of CLAD lungs identified increased activator protein-1 (AP-1)-related programs, including JUNB and FOS, in both epithelial and endothelial compartments, together with inflammatory and fibrotic networks converging on TNF and IL-17 signaling (54). In BOS-type CLAD, airway epithelial cells also exhibited an interferon regulatory factor 7 (IRF7)-centered interferon response together with fibrosis-associated programs. Banday and colleagues functionally linked this airway-centered molecular program to fibrotic remodeling using primary bronchial epithelial cells cultured at an air–liquid interface and human precision-cut lung slices. Influenza-induced activation of IRF7 promoted fibrotic responses through IL-33 and MMP-9, whereas inhibition of these pathways reduced α-smooth muscle actin (α-SMA) expression and collagen accumulation (55).

These findings are important because they move beyond spatial association to connect an airway-centered molecular program with a functional injury-to-fibrosis circuit. They further support a view of CLAD not as an isolated macrophage abnormality, but as coordinated reorganization of a local tissue network spanning epithelial, immune, vascular, and stromal compartments. Such local tissue responses are also heterogeneous within individual lungs. Large-scale imaging-based transcriptomic profiling across BOS, mild RAS, and advanced fibrotic RAS identified varying contributions of epithelial stress and innate immunity, adaptive immunity and cytotoxicity, ECM remodeling, and endothelial dysfunction, together with pronounced molecular heterogeneity among tissue regions from the same patient (56).

Taken together, these studies support a model in which repeated airway epithelial injury in post-lung-transplant BOS is accompanied by restructuring of the resident macrophage compartment, recruitment of recipient-derived immune cells, and vascular and stromal responses, which may collectively contribute to the establishment and persistence of a pathological cellular niche on the anatomical scaffold of the small airway. In this framework, disease is not determined by a particular macrophage phenotype alone, but by the origin and state of macrophages in relation to their anatomical location and the neighboring cells with which they interact.

### 6.2 BOS after hematopoietic stem cell transplantation

BOS after hematopoietic stem cell transplantation (HSCT) is a major pulmonary manifestation of chronic graft-versus-host disease (cGVHD) and is characterized by progressive, fixed airflow obstruction (57). Mechanistic studies of HSCT-associated BOS were substantially advanced by the development of murine models that reproduce pulmonary dysfunction, peribronchiolar inflammation, and airway obliteration after allogeneic bone marrow transplantation (58). Subsequent studies have established that BOS is not simply a consequence of T-cell-mediated injury, but rather reflects a stepwise and interdependent process linking adaptive immune responses to tissue fibrosis. In particular, class-switched alloantibodies derived from donor B cells, germinal-center formation, and the T follicular helper (Tfh) cell–germinal-center B-cell axis contribute to disease, and disruption of these pathways attenuates pulmonary dysfunction, immunoglobulin deposition, and collagen accumulation (59, 60).

The monocyte/macrophage compartment becomes particularly important during the fibrotic effector phase that follows this adaptive immune dysregulation. In mice with established BOS-like cGVHD, pirfenidone treatment reduced the accumulation of peribronchiolar F4/80+ macrophages and transforming growth factor-β (TGF-β) and improved both collagen deposition and pulmonary dysfunction (61). Similarly, IL-17A blockade reduced CCL2 expression, recruitment of inflammatory monocytes and macrophages, and airway and parenchymal fibrosis, supporting a pathway through which inflammatory lymphocyte responses are coupled to monocyte/macrophage recruitment and subsequent fibrotic remodeling (62).

Recent single-cell studies have further resolved this transition from immune activation to fibrosis at the level of cellular states. In lungs from murine BOS cGVHD, both alveolar and interstitial macrophages exhibited profibrotic programs that included Arginase1 and Tgfb1. Bromodomain and extraterminal inhibition simultaneously suppressed Tfh/germinal-center B-cell responses and pulmonary immunoglobulin deposition while reducing CD206+ Fc gamma receptor (FcγR)+ macrophages and profibrotic macrophage states (63). These findings suggest that adaptive immune activation and macrophage-dependent fibrosis do not proceed as independent processes in HSCT-associated BOS, but rather form interconnected components of a multicellular pathological network.

In contrast to this relatively detailed preclinical framework, spatial evidence from human HSCT-associated BOS has remained limited. Yamamoto and colleagues analyzed explanted lungs from patients undergoing lung transplantation for severe HSCT-associated BOS/bronchiolitis obliterans and identified accumulation of foamy macrophages within and around obliterated bronchioles (64). GeoMx profiling demonstrated enrichment of macrophage-related genes and TGF-β signaling in these regions, together with a shift from inflammation-associated to fibrosis-associated programs according to histological stage and anatomical location. Foamy macrophages coexpressing CD68, CD206, and α-SMA also accumulated around obliterated bronchioles, displaying a mixed-marker phenotype compatible with macrophage-to-myofibroblast transition (MMT) (64). Coexpression of CD68/CD206 and α-SMA in human tissue, however, does not by itself establish lineage conversion from macrophages to myofibroblasts. These observations are therefore more appropriately interpreted as evidence that cells with an MMT-compatible phenotype accumulate spatially within BOS lesions and that cellular states positioned at the interface between macrophage and fibroblast phenotypes may contribute to airway fibrosis.

The direction of alloreactivity is opposite in lung-transplant and HSCT-associated BOS. In lung transplantation, the recipient immune system targets the donor-derived lung allograft, whereas after HSCT, donor-derived immune cells target recipient airway tissues. Despite this fundamental difference, both conditions converge on repeated epithelial injury, adaptive immune dysregulation, monocyte/macrophage recruitment, and profibrotic responses involving TGF-β, with these processes ultimately concentrated around the small airways. This convergence suggests that the pathological architecture of fibrosis may be shaped not only by disease etiology, but also by the anatomical scaffold on which pathological cellular states are assembled and by the local cellular relationships through which they may become stabilized.

## 7 Therapeutic strategies targeting spatial niches

An important implication emerging from spatial analyses across diseases is that therapeutic targeting may be extended beyond the “pathological macrophage” as a cell population to the local intercellular interactions that establish and sustain its pathogenic state. In models of pulmonary fibrosis, selective depletion of monocyte-derived alveolar macrophages (Mo-AMs) attenuates fibrosis, whereas depletion of tissue-resident alveolar macrophages does not produce the same effect, underscoring the importance of distinguishing macrophage origin and state when designing therapeutic interventions (44). Translating such selective approaches to human disease, however, will require reliable discrimination of disease-associated macrophage populations from macrophages that maintain normal tissue homeostasis.

A more direct therapeutic opportunity may lie in targeting the intercellular signals that sustain pathological niches. The post-viral T cell–macrophage–epithelial circuit discussed above illustrates this principle, as blockade of its key inflammatory signals improved alveolar regeneration and lung function (12). Similarly, in BOS after lung transplantation, an IRF7-associated airway epithelial program identified by GeoMx profiling was functionally validated in human airway epithelial cultures and precision-cut lung slices as an IRF7–IL-33–MMP-9 axis (55). Together, these studies illustrate a practical route toward niche-informed therapeutic target discovery: spatial profiling identifies candidate pathological niches, and functional perturbation defines the molecular circuits required for their maintenance.

Future studies should determine whether pathological cellular relationships can be disrupted not only by controlling cellular recruitment and local persistence, but also by modifying the surrounding tissue environment, including the ECM. The ultimate goal should not be indiscriminate depletion of a given cell population, but selective disruption of pathological niches while preserving, or ideally restoring, the cellular communities that support normal tissue homeostasis.

## 8 Discussion

Integrating the evidence from acute lung injury and chronic fibrosis discussed above reveals several recurring spatial principles that may provide a useful conceptual framework for understanding these conditions. These include injury-induced reorganization of the resident macrophage compartment and its niche, pathological persistence of normally transient cellular states, stabilization of these states in association with, and in some settings through, interactions with neighboring cells, and the influence of anatomical location on the ultimate tissue architecture. Within this framework, loss of tissue-resident macrophages, recruitment of monocyte-derived cells, persistence of regenerative epithelial intermediates, and activation of stromal populations can become interconnected within local pathological niches. However, although this framework is useful for understanding defective repair and fibrosis, not all macrophage-centered spatial organizations can be explained as manifestations of failed repair. Granulomatous disease and lung cancer provide important comparisons through which to define both the generalizability and the limits of this model.

### 8.1 Granulomas: macrophage-centered spatial organization beyond failed repair

Granulomas are organized tissue responses to pathogens or persistent antigens that are difficult to eradicate, in which macrophage-centered cellular communities act to spatially contain the inciting stimulus (65). Thus, whereas fibrotic niches are primarily associated with tissue remodeling and replacement of normal architecture, the central spatial function of a granuloma is compartmentalization of a persistent stimulus. These processes are not mutually exclusive, however, as chronic granulomatous lesions can also harbor fibrotic programs within the same tissue environment.

MIBI-TOF analysis of human tuberculosis granulomas has shown that multiple immune-cell populations are organized into functionally distinct spatial microenvironments, with regions of immune activation and immune regulation coexisting within individual granulomas (66). These findings challenge a simplified concentric model in which granulomas consist only of a macrophage-rich core surrounded by peripheral lymphocytes.

Spatial transcriptomic analysis of chronic pulmonary sarcoidosis further illustrates this complexity. Macrophages expressing SPP1, CHIT1, and CHI3L1 accumulated within granuloma cores and displayed transcriptional features associated with both inflammation and fibrosis, whereas peripheral regions contained fibroblast niches enriched for collagen and CTHRC1 expression (67). Thus, granulomatous inflammation and fibrosis share the broader principle of persistent multicellular organization involving macrophages, yet differ in their primary spatial function and histopathological outcome. This comparison reinforces the idea that disease cannot be defined by a particular macrophage phenotype alone, but instead depends on the composition and spatial organization of the niche as a whole.

### 8.2 Lung cancer: spatial divergence of macrophage function

Lung cancer extends this principle further. Large-scale imaging mass cytometry of lung adenocarcinoma has shown that not only the abundance of immune cells, but also the composition and spatial organization of cellular neighborhoods containing macrophages, T cells, endothelial cells, and tumor cells are associated with histopathological features and clinical outcome (68). Tissue-resident macrophages also localize near tumor cells during early lung tumorigenesis, where they promote tumor-cell invasiveness and regulatory T-cell responses, while their spatial distribution changes as tumors progress (69). These observations indicate that macrophages of the same lineage can acquire distinct functions depending on both disease stage and spatial context.

Particularly relevant to fibrosis is the observation that lung tumors also exploit tissue-remodeling programs involving macrophages, fibroblasts, and ECM. In non-small-cell lung cancer (NSCLC), SPP1+ macrophages and COL11A1+ cancer-associated fibroblasts colocalize at the tumor boundary, where an ECM-remodeling, T-cell-excluding microenvironment is associated with resistance to therapy (70). Similarly, regions enriched in SLC2A1+ tumor-associated macrophages (TAMs) exhibit impaired CD8+ T-cell function and are associated with resistance to immunotherapy, suggesting that the metabolic state of TAMs can also shape local immune function (71).

Importantly, however, not all TAM-containing niches are immunosuppressive. In human lung cancer, a localized stem-immunity hub composed of stem-like CD8+ T cells, mature dendritic cells, fibroblasts, and CXCL10+ macrophages has been identified and associated with favorable responses to programmed cell death protein 1 (PD-1) blockade (72). In lung cancer models, peribronchovascular interstitial macrophages expressing CXCL9, CXCL10, and CXCL13 support lymphocyte recruitment and tumor control, whereas intratumoral CCL2+ macrophages recruit monocyte-derived cells and promote a tumor-supportive environment (73).

Lung cancer therefore cannot be reduced to a “wound that does not heal” or viewed simply as an extension of the fibrotic niche. Rather, tissue-remodeling programs also used during repair and fibrosis are repurposed and reorganized in tumors under the additional pressures of tumor-cell clonal evolution, metabolic competition, and therapeutic selection. Pan-cancer analyses showing that the relative expression of CXCL9 and SPP1 reflects coordinated TAM-associated cellular programs further support this functional continuum (74). The lung cancer setting therefore provides a particularly clear example of why macrophage function should not be inferred from a simple M1/M2 classification or a single marker, but instead evaluated in the context of cellular origin, state, anatomical location, and interactions with neighboring cells.

### 8.3 Limitations of spatial analysis: from proximity to causality

Spatial omics provides a powerful means of studying cellular states within intact tissue architecture, but it is subject to important technical and interpretative limitations. Conventional spot-based spatial transcriptomics offers broad transcriptomic coverage, yet measurement units containing multiple cells limit precise assignment of cell types and transcripts. By contrast, high-resolution *in situ* platforms such as Xenium and CosMx provide single-cell and, in some cases, subcellular spatial information, but generally rely on predefined gene panels and therefore constrain discovery of unanticipated molecular programs (3, 6). Spatial datasets also possess distinctive statistical properties, including spatial autocorrelation and dependence on analytical scale, such that the definition of cellular neighborhoods and the apparent strength of spatial associations can vary according to the analytical framework used (75).

A more fundamental limitation is that spatial proximity cannot be equated with functional interaction. Cellular proximity or colocalization, as well as concordant ligand–receptor expression, can nominate candidate cell–cell interactions but do not establish their directionality or causal contribution to disease (32). Similar caution is warranted when inferring temporal progression from spatial gradients across distinct lesion states or lineage conversion from coexpression of markers associated with different cellular lineages. Similarly, in human spatial datasets, transcriptional phenotype and anatomical localization may suggest macrophage identity or origin but do not directly establish ontogeny. A spatially defined “niche” should therefore initially be regarded as a candidate unit of disease organization rather than a demonstrated causal mechanism.

The central challenge ahead is therefore not simply to achieve ever-higher spatial resolution, but to connect observed spatial structures to causal disease mechanisms. Integrating spatial omics from patient tissues with lineage tracing, longitudinal analysis, genetic and pharmacological perturbation, and functional models such as organoids, co-culture systems, and precision-cut lung slices will be essential for distinguishing the intercellular interactions that are truly required to sustain disease from the many associations revealed by spatial profiling. Ultimately, the value of spatial analysis lies not merely in describing which cells are located where, but in identifying which spatial relationships maintain disease and how those relationships can be translated into actionable mechanisms.

## 9 Conclusion

Advances in spatial analysis are redefining lung macrophage function as a property not of a fixed cellular phenotype, but of cellular origin, state, anatomical location, and relationships with neighboring cells. Across acute lung injury and chronic fibrosis, failure to resolve normally transient repair-associated niches and persistent spatial stabilization of multicellular interactions may contribute to disease maintenance, whereas the ultimate histopathological outcome may be shaped in part by the anatomical compartment in which these niches are established. Moving forward, linking spatial proximity to functional causality and selectively targeting the local niches that sustain pathological states, rather than the pathological cells alone, may provide a path toward both deeper mechanistic understanding and new therapeutic strategies for lung disease.
